# Subclavian Vein Thrombosis Extending into the Internal Jugular Vein: Paget-von Schroetter Syndrome

**DOI:** 10.4021/jocmr2009.07.1248

**Published:** 2009-08-20

**Authors:** Enver Ilhan, Mehmet Ture, Cengiz Yilmaz, Muhammed Arslan

**Affiliations:** a Department of General Surgery, Izmir Bozyaka Education and Research Hospital, Izmir, Turkey; b Department of Cardiovascular Surgery, Izmir Bozyaka Education and Research Hospital, Izmir, Turkey; c Department of Radiology, Izmir Bozyaka Education and Research Hospital, Izmir, Turkey

## Abstract

Paget-von Schroetter syndrome refers to spontaneous thrombosis of the subclavian vein and constitutes 0.5-1% of all venous thromboses. It is prevalent among young and healthy adult males who engage in sports. Early diagnosis and treatment is essential to avoid long-term sequelae. Herein, we report a 42-year-old male weightlifter who presented with swelling of the left arm, pain and a feeling of tightness in the anterior chest wall. At Doppler US and MR angiography, subclavian vein thrombosis extending into the internal jugular vein was detected. Thrombolytic treatment ensured continuous venous flow and the patient’s complaints decreased.

**Keywords:**

Veins; Thrombosis; Thorombolytic therapy

## Introduction

Effort thrombosis or Paget-Schroetter Syndrome (PSS) most often develops among young adults engaging in sport activities and those who work in jobs that require repeated arm movements which cause axillo-subclavian vein trauma and facilitate the development of deep vein thrombosis [1]. It frequently occurs as a result of the chronic compression of the subclavian vein at the thoracic outlet level, the costoclavicular junction [2, 3]. With a high prevalence among young people and active adults, this syndrome has a considerably high probability of morbidity in later decades unless it is diagnosed on time and properly treated [1]. In this report, we aimed to underline the importance of early diagnosis and treatment for this rare disease.

## Case Report

**Figure 1:**
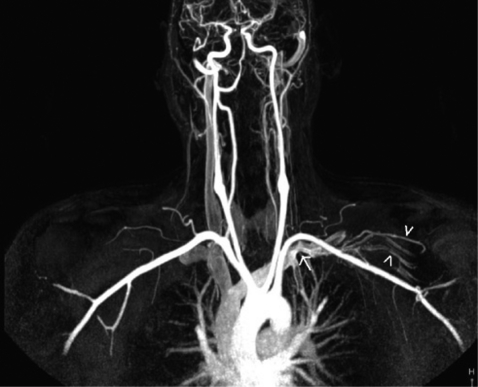
Coronal maximum-intensity-projection contrast-enhanced MR angiogram obtained after left antecubital vein injection reveals near-complet occlusion of the left proksimal subclavian vein (arrow) and distal venous collaterals( arrowhead).

**Figure 2:**
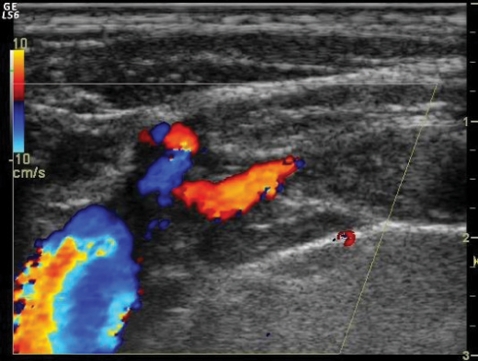
Longitudinal color Doppler sonogram reveals near complete thrombotic occlusion of the left subclavian vein.

**Figure 3:**
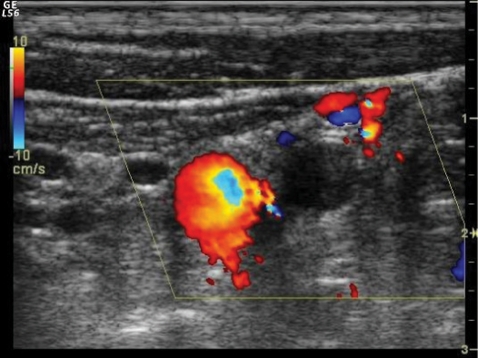
Transverse color Doppler image shows extension of the thrombotic material into the proximal part of the internal jugular vein.

**Figure 4:**
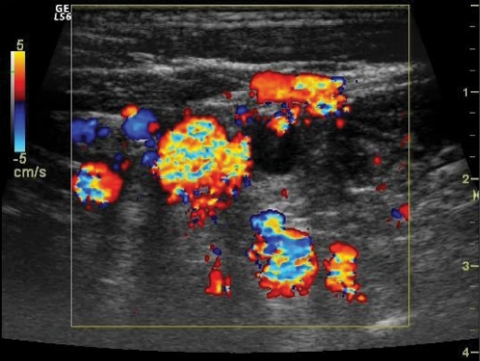
Transverse color Doppler image demonstrates prominent cervical venous collateralisation.

A 42-year-old male presented with pain and swelling of the left arm after a sequence of intense, repetitive weightlifting exercises. Upon questioning, he disclosed that he had been engaged with weightlifting for a long time and had complaints for a while. His medical history was unremarkable. Laboratory tests were positive for antithrombin 3 and factor leiden, agents that facilitate coagulation. Based on these findings, upper-extremity effort thrombosis was suspected. Thereafter, contrast enhanced upper-extremity venous MR angiography (MRA) and color Doppler US (CDUS) were performed to verify the diagnosis. Contrast-enhanced MRA revealed near-complete occlusion of the proximal left subclavian vein and distal collateral formations (Fig. 1). CDUS showed a heterogeneous thrombotic mass that filled almost the entire proximal segment of the left subclavian vein (Fig. 2). Thrombosis extended into the proximal segment of the left internal jugular vein (IJV) (Fig. 3). Furthermore, extensive venous collateral formations were presented in the left proximal cervical localization (Fig. 4). Both MR angiographic and sonographic findings were consistent with PSS.

As the patient had already developed extensive venous collaterals, no surgical intervention was performed. Instead, treatment with low-molecular weight heparin and anticoagulants, was initiated and continued along with the follow-up for bleeding parameters. As of 3 years clinical follow-up, the patient is doing well and the treatment is continued with oral anticoagulants and acetylsalicylic acid.

## Discussion

Spontaneous thrombosis in the upper-extremity veins was first described by Sir James Paget in 1875. Later, in 1884, Von Schroetter associated this condition with thrombotic occlusion of the axillary vein and the subclavian vein [4].

Spontaneous or effort-related thrombosis of the axillo-subclavian vein is referred to as PSS, the disease of actively working young men. Around 75% of the cases usually occur in the dominant upper extremity after extraordinary arm position or exercise. Shoulder movements aggravate the complaints [5, 6]. Although it is more frequent among male athletes, today, it also occurs among women engaging in sport activities [1]. These patients are likely to have lowered living standards in the later years of their life with disease sequelae continuing throughout their lives [7].

PSS should be considered in all young patients actively working, engaging in sports and presenting with unilateral swelling of the arm. Patient histories often include using one arm with frequently repeated movements. Usually, the dominant arm is affected [7].

In our case, the thrombotic material was shown to extend into the proximal left internal jugular vein. To our knowledge, extension of subclavian venous thrombosis thorough the IJV in PSS is exceptionally rare and has been reported only in one recently published article [8].

Early diagnosis is crucial for rapid venous recanalization through anticoagulant treatment.

Indications for surgical treatment in PSS remain controversial. As soon as early diagnosis is available, a multidisciplinary approach is needed [4]. Treatment methods might change with personal, institutional, and regional preferences.

Treatment involves surgical procedures such as catheter-directed subclavian vein thrombolysis, balloon angioplasty, stent placement, paraclavicular thoracic outlet decompression, saphenous vein patch angioplasty, reversed saphenous vein graft bypass, rib resection, scalenectomy, resection of the clavicular callus, and axillo-jugular bypass. Aggressive endovascular treatment is also effective [1, 2, 6, 7, 9, 10]. In our case, surgical treatment was not deemed to be appropriate as the patient had already developed extensive venous collaterals.

In conclusion, PSS should be considered in the differential diagnosis of effort induced upper extremity pain and swelling. Conservative non-operative treatment is acceptable and can be successfully used with favorable long-term outcomes.
